# Resuscitation and auto resuscitation by airway reflexes in animals

**DOI:** 10.1186/1745-9974-9-21

**Published:** 2013-08-22

**Authors:** Zoltan Tomori, Viliam Donic, Roman Benacka, Jan Jakus, Sona Gresova

**Affiliations:** 1 Department of Human Physiology Faculty of Medicine, University of PJ Safarik, Kosice, Slovakia; 2 Department of Pathophysiology, Faculty of Medicine, University of PJ Safarik, Kosice, Slovakia; 3 Jessenius Faculty of Medicine in Martin, Comenius University, Bratislava, Slovakia

**Keywords:** Animals, Asphyxia, Aspiration reflex, Autoresuscitation, Cough, Expiration reflex, Resuscitation

## Abstract

Various diseases often result in decompensation requiring resuscitation. In infants moderate hypoxia evokes a compensatory augmented breath – sigh and more severe hypoxia results in a solitary gasp. Progressive asphyxia provokes gasping respiration saving the healthy infant – autoresuscitation by gasping. A neonate with sudden infant death syndrome, however, usually will not survive. Our systematic research in animals indicated that airway reflexes have similar resuscitation potential as gasping respiration. Nasopharyngeal stimulation in cats and most mammals evokes the aspiration reflex, characterized by spasmodic inspiration followed by passive expiration. On the contrary, expiration reflex from the larynx, or cough reflex from the pharynx and lower airways manifest by a forced expiration, which in cough is preceded by deep inspiration. These reflexes of distinct character activate the brainstem rhythm generators for inspiration and expiration strongly, but differently. They secondarily modulate the control mechanisms of various vital functions of the organism. During severe asphyxia the progressive respiratory insufficiency may induce a life-threatening cardio-respiratory failure. The sniff- and gasp-like aspiration reflex and similar spasmodic inspirations, accompanied by strong sympatho-adrenergic activation, can interrupt a severe asphyxia and reverse the developing dangerous cardiovascular and vasomotor dysfunctions, threatening with imminent loss of consciousness and death. During progressive asphyxia the reversal of gradually developing bradycardia and excessive hypotension by airway reflexes starts with reflex tachycardia and vasoconstriction, resulting in prompt hypertensive reaction, followed by renewal of cortical activity and gradual normalization of breathing. A combination of the aspiration reflex supporting venous return and the expiration or cough reflex increasing the cerebral perfusion by strong expirations, provides a powerful resuscitation and autoresuscitation potential, proved in animal experiments. They represent a simple but unique model tested in animal experiments.

## Background

Breathing can be frequently modified reflexly or voluntarily. According to time and intensity characteristics, the modifications of breathing can be well assessed by recording of electromyogram (EMG) of inspiratory and expiratory muscles and airflow, as well as the activity of afferent and efferent nerves and their central structures. Monitoring of breathing and other physiological parameters in infants indicated that apnoeic episodes and occasional occlusion of the face-mask outlet evoke 4 different types of reaction. They depend mostly on the intensity of the resulting hypoxaemia and hypercapnia, as well as on the maturity of the infants’ cardio-respiratory control mechanisms. Polysomnography in healthy infants during sleep indicated, that an occasional airway occlusion, causing hypoxia usually evoked a startle reaction, accompanied by limb and nuchal EMG activation, neck extension, and heart rate (HR) acceleration. There was a simultaneous large biphasic inspiratory effort - *augmented breath or sigh*, where the intensity of startle correlated with the magnitude of maximal negative airway pressure and HR acceleration. These results indicate that the augmented part of sigh coincided with the genio-glossal (GG) muscle activation, resulting in frequent opening of airway closure with only brainstem or sub-cortical mechanism, but without cortical involvement. More severe hypoxia results in *a solitary gasp*. These reactions improved with age and were not caused by stimulation of stretch receptors due to lung inflation [1-3].

**Figure 1:**
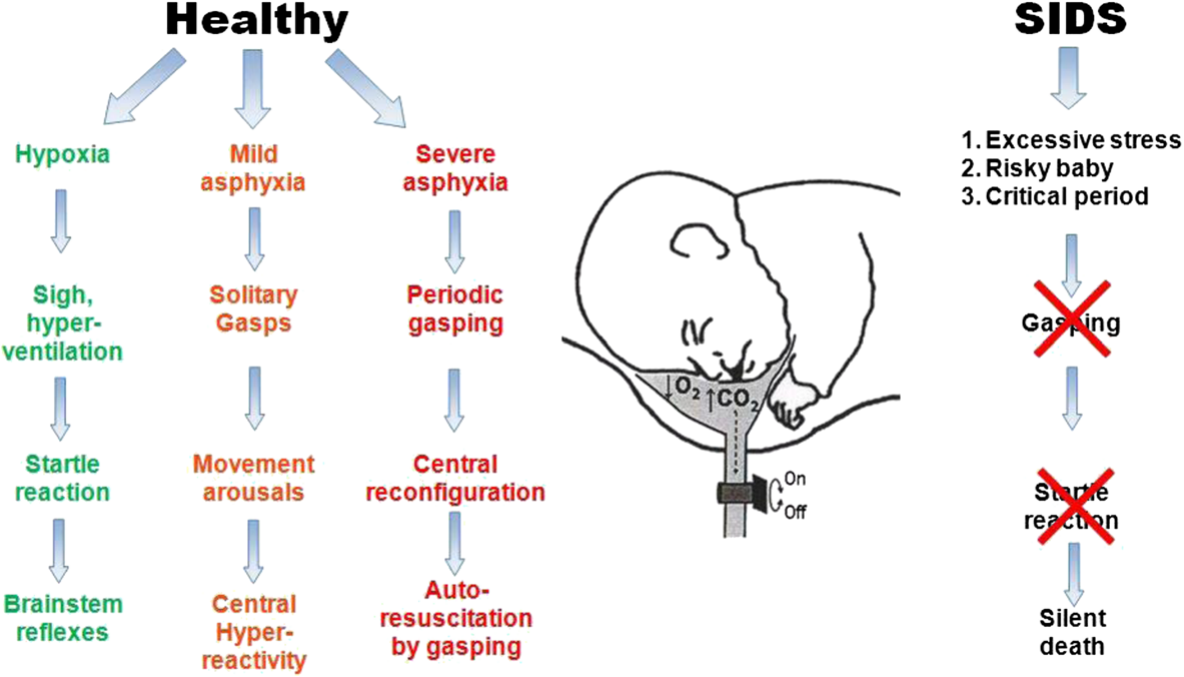
**Autoresuscitation by gasping in healthy infant and its failure in neonate with SIDS.** In a normal infant an inhalation of lower O_2_ and/or higher CO_2_ content in air evokes hypoxia with occurrence of solitary sighs or gasps. The sigh is accompanied by a startle reaction, which manifests with neck extension and upper airway dilation, resulting in normalization of breathing. Severe hypoxia provokes solitary gasps accompanied by cortical arousal and movements. Progressive asphyxia provokes a period of gasping respiration contributing to autoresuscitation by gasping. Similar asphyxia in a risky preterm baby (including SIDS during a critical period after birth), does not evoke solitary sighs with startle or gasps, but results in silent death. Reproduced with permission and modified from [31].

Gasp as a primitive form of breathing develops during the foetal life in mammals. As the first breaths after birth connected with hypoxia, sighs and solitary gasps tend to distend the atelectatic alveoli, contributing to a gradual distension of the lungs in newborns. Stronger and longer-lasting asphyxia after reconfiguration of the cardio-respiratory control mechanisms provokes development of *gasping respiration*. This is characterized already by markedly depressed brain function with flat electroencephalogram (EEG), suppressed peripheral reflexes and muscular atonia. Gasping respiration develops often before death as a last resort, tending to restore the failing vital functions and to resuscitate the mammals - *autoresuscitation by gasping*[4-9]. This autoresuscitation mechanism may be unsuccessful in excessive and long-term asphyxia, or in babies with under-developed cardio-respiratory control mechanisms. Such a failure may occur particularly during the first months after birth, often resulting in *silent death* (Figure 1). Therefore, the explanation of the mechanisms of autoresuscitation by gasping appeared to be extremely important, particularly in infants.

### Mechanisms of autoresuscitation by gasping in animals and infants

Gasping respiration is a critical mechanism for survival and it serves for autoresuscitation in all mammalian species from the day of birth, when eupnoea fails. Autoresuscitation by gasping represents a very effective life-saving mechanism from severe cardio-respiratory failure, accompanied by deep coma. The effects of this primitive type of breathing were tested in experimental models in pigs, cats and other animals. Piglets were studied to determine the cardiovascular and neuro-physiological effects of prolonged laryngeal-induced respiratory inhibition. During continuous laryngeal stimulation in light anaesthesia the EEG becames flat by 1 min after the onset of apnoea and remained iso-electric throughout the stimulation period. Apnoea was interrupted every 1–2.5 min by clusters of 2–6 gasp-like breaths. With each cluster of gasps, arterial PO_2_ and mean blood pressure (BP) increased. These data indicated that despite EEG silence, *piglets can autoresuscitate from asphyxia by initiating gasping*, which may markedly improve the cardiovascular status, and sustain animals for a prolonged period of time [6]. In animal study with untreated experimental ventricular fibrillation (VF) and postponed defibrillation, gasping developed gradually during 4–6 minutes, transiently providing sufficient venous return of blood to the heart and a continual perfusion of the brain and other vital organs. In such a manner *gasping respiration provided cardiac output during cardiac arrest* and saved certain animals according to the reserves of their vital functions [10].

Many mammals are born immature and in addition to retardation in physical and musculoskeletal growth, several patho-physiological defects including the cardio-respiratory responses to hypoxia and hypothermia may also occur. In an in vitro preparation, endogenous 5-hydroxy tryptamine (5-HT) is reported to be essential for expression of gasping. Using an in situ preparation of the Pet-1 knockout mouse, the number of 5-HT neurons is reduced by 85-90% compared with animals without this homozygous genetic defect. Despite this reduction in the number of serotoninergic neurons, phrenic discharge in eupnoea and gasping of Pet-1 knockout mice was not different from that of wild-type mice. Gasping continued unabated, even after administration of methysergide, a blocker of many types of receptors for 5-HT, indicating independence of gasping on levels of serotonin [11]. On the other side, HR recovery failed at a critical age in 5 HT deficient mice exposed to episodic anoxia [12].

*The neuro-genesis of gasping* is dependent on the discharge of neurons in the rostro-ventral medulla, overlapping “the pre-Bötzinger complex” (preBötC). Neuronal activities of this complex, characterized in an in vitro brainstem-spinal cord preparation of the neonatal rat, have been hypothesized to underlie respiratory rhythm generation. The rhythmic activity of this in vitro preparation is markedly different from eupnoea, but identical with gasping in vivo. Medullary neuronal activities generating the gasp and the identical rhythm of the in vitro preparation are incorporated into the ponto-medullary circuit defining ventilatory activity [13].

EMG activity of upper airway (UA) genio-glossal muscle -GG and the diaphragm (D) were studied in anaesthetized rabbits during progressive asphyxia induced by airway occlusion. Peak activity of GG increased more than that of the D during hyperpnoea and gasping. These data indicate differences in the control mechanism of the GG and D during acute severe asphyxia. Increased UA muscle activity seen during gasping should help preserve UA patency, and facilitate autoresuscitation by gasping. These observations support the concept that *gasping is a highly organized function of the respiratory center*[14,15]. During severe hypoxia the network properties within the preBötC are reconfigured, whereby it no longer generates eupnoea, but instead generates gasping. Such reconfiguration includes changes in synaptic and intrinsic properties triggered by hypoxia itself, as well as the influence of different neuromodulators released during hypoxia. Therefore, gasping respiration has been considered an important mechanism, that triggers autoresuscitation. Deregulation of gasping has been proposed to result in failure to autoresuscitate and has been hypothesized to contribute to development of SIDS [7,8]. Precisely which synaptic and/or neuronal intrinsic membrane properties are critical to central respiratory rhythmogenesis, in either normoxia or hypoxia, is still the subject of considerable discussion [13-16].

### Protective and defensive airway reflexes

**Figure 2:**
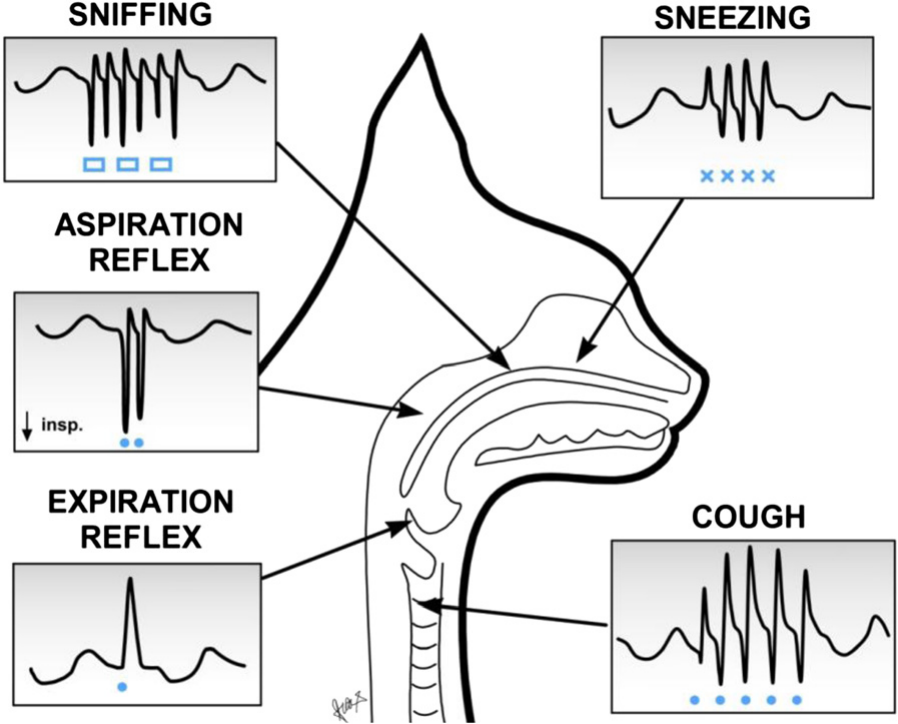
**Schematic presentation of the main protective and defensive reflexes in cats.** Airflow record (V’) with inspiration downward (↓). Stimulation of specific airway areas using mechanical contacts with a nylon fiber (-----) and presentation of odorous substances (**□□□**) are indicated in anaesthetized cat. In addition to gasp- and sniff-like aspiration reflex elicited from the nasopharynx, sniffing from the upper part of nasal cavity, expiration reflex from the larynx, the cough reflex from the trachea-bronchial region and sneezing from the nasal mucosa. Modified from our earlier publications [22,31,40].

From several reflexes evoked by stimulation of various areas of the airways, the following three, illustrated in Figure 2, are especially important for the protection and defence of the respiratory system. *A gasp-like aspiration reflex (AspR)* can be regularly evoked by various mechanical, electrical, and other methods of stimulation of the nasopharynx (NPh) in both anaesthetized and non-anaesthetized cats and other mammals. It manifests as a solitary spasmodic inspiration (SI) that only lasts for 150–230 ms, and for its tendency to inhibit expiratory efforts it is usually not followed by an immediate active expiration [17-21]. However, the irritant substances may be transported by the rapid and strong inspiratory airflow, and a muco-ciliary transport from NPh to the hypopharynx, from where they may provoke reflex swallowing or cough. Accidental input of irritants into the larynx usually provokes laryngo-constriction and the *expiration reflex (ExpR)* or a prompt *cough reflex (CR)*, providing expulsion of irritants, and so preventing their aspiration into the lungs [22]. The tendency to provoke SIs is high at the beginning of spontaneous inspiration for ventilatory drive, but it gradually decreases during the inspiratory phase [23]. The AspR can be induced also by negative or positive pressure air puff stimulation of the upper airways [24]. In paralyzed cats, stimulation of the „irritant” rapidly adapting receptors (RARs) of the NPh by mechanical contacts and pressure pulses or air-puffs provokes a very strong activity in the glossopharyngeal afferent fibers, resulting in AspR. The frequency of afferent impulses is very high (mean 197/s, range 67-330/s) [25], which is >10 times higher than during control inspiration. They strongly activate many brainstem inspiratory neurons, including “the inspiratory pattern generator”, described in the brainstem of juvenile rats [26], and the respiratory central pattern generator (CPG), was analyzed more in detail in several papers [12,27,28]. Strong activation of various inspiratory neurons of adult cats by AspR was identified by c-Fos immuno-reactive method in 14 of 35 tested brainstem nuclei [29].

**Figure 3:**
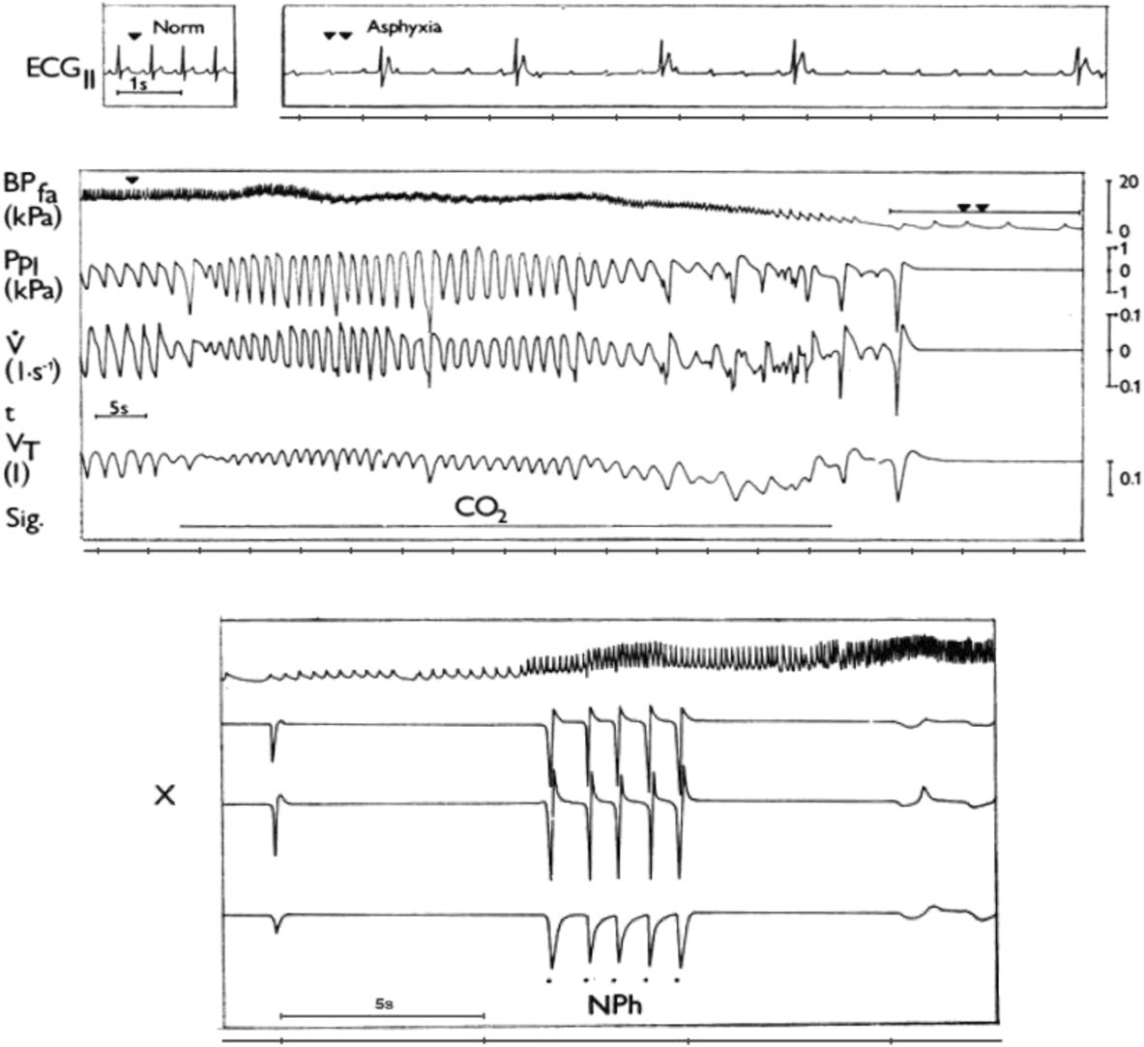
**Cardio-respiratory failure in anaesthetized cat during asphyxia and resuscitation by AspR.** Cardio-respiratory changes in anaesthetized cat during asphyxia caused by inhalation of CO_2_ and resuscitation by AspR provoked by mechanical stimulation of nasopharynx (NPh). Recordings, from above: ECG_II_- electrocardiogram, BP_fa_- blood pressure in femoral artery, P_pl_ – pleural pressure, V**´** – tracheal airflow, V_T_ – tidal volume, Sig. – signal for administration of CO_2_ or stimulation of nasopharynx, x – continuation of recording, t – time indicated in intervals of 5 seconds. Reproduced with permission and modified from [18].

***The AspR*** is characterized by a specific time-frequency distribution of powerful energy in the phrenic nerve, manifesting with high frequency oscillations [30]. Such powerful activation of the “presupposed inspiratory generator” by the AspR [31] can very effectively modify (facilitate or inhibit through dense synaptic connections [32] and several mediators the “central mechanisms” of various vital functions [31]. Recording and power spectral analysis of the phrenic and the hypoglossal nerve activities in paralyzed cats, indicated very similar character and peaks during both the hypoxic medullary gasping and the AspR evoked by mechanical stimulation of NPh in normoxic conditions [33,34]. Such close similarity of AspR with gasping suggests their similar resuscitation potential, resembling autoresuscitation of human infants by gasping [4,5,7]. Great similarity and vitality of AspR with gasping was supported by its persistence at the absence of CR and ExpR in premortal gasping stage after medullary transsection 5 mm above the obex in cats [35], fitting the localization of “inspiratory generator”. The resuscitation potential of AspR was proved by termination of progressive hypotension and atrio-ventricular (A-V) blockade in a cat, during gasping stage caused by severe asphyxia, indicated on Figure 3, analyzed later more in detail. Similar gasp-like inspirations were elicited by mechanical stimulation of NPh also in adult rats [36], dogs [37] and premature infants [38], as well as by inflation of the whole respiratory system also in newborn babies [39]. Such approved similarity of gasp-like AspR in cats and other animals, with the gasping in infants suggests also similar resuscitation potential for AspR in cats and other animals, than gasping in human infants. It allows also a hypothesis that normalization or resuscitation and even autoresuscitation potential of airway reflexes may exist in humans, which was supported by reversal of various functional disorders using airway reflexes in men [40].

***The cough reflex*** induced by stimulation of various airway receptors, held as a “watchdog of the lung” consists of 3 phases. After the initial *deep inspiration (DI)* there is a rapid compressive phase with glottal closure, followed by strong expulsion of the enlarged lung volume [21,22,31,40,41]. The gradually increasing lung volume stimulates the SARs, and via the Hering- Breuer inflation reflex (HBIR) tends to inhibite adequately the progressive inspiration similarly as in eupnoea. The expiratory effort is very strong at the beginning of the expiratory phase of CR due to Hering Breuer expiratory facilitation reflex (HBEFR), caused by enlarged lung volume [23]. Therefore, *the deeper the initial DI, the more powerful or effective is usually the subsequent expiratory effort.* During a series of cough efforts persisting after the end of stimulation, their intensity gradually decreases with the lowering of lung volume, ventilatory drive, peak expiratory flow, and with prolongation of the second non-active part of the expiratory phase of cough. In non-paralyzed subjects, the myotatic reflexes of the respiratory muscles and stimulation of various thoracic and abdominal proprioceptors, as well as irritant RARs and C fibres in the airways and lungs, caused by flow and volume changes, can also contribute to the modification of the successive cough efforts [31,41]. Somato-sensory nerves innervating the chest wall, D and abdominal muscles, as well as nerves of visceral organs also likely play important role in regulating cough [42]. Cough is not a stereotyped output from the medullary “*cough center*”, but its pattern and strength depend on many afferent inputs on the “cough center” [43].

Experiments in paralyzed cats indicated close functional connectivity of ventro-lateral medullary neurons with phrenic, lumbar and recurrent laryngeal nerves during *fictive coughing*, induced by mechanical stimulation of the intrathoracic trachea. During the inspiratory phase, excitation of medullary inspiratory augmenting (IAug) neurons, are connected with activation of phrenic and recurrent laryngeal nerves. In expiratory phase, the activity of expiratory augmenting (EAug) neurons, are accompanied by activation of the lumbar and laryngeal nerves [27]. *The CPG undergoes reconfiguration to produce cough.* About 1/3 of medullary inspiratory decrementing (IDec) neurons of the *caudal ventral respiratory column (cVRC),* during the first cycle (C_1_) of cough attack changes to IAug type. This is accompanied by increases of phrenic activity, esophageal pressure and the inspiratory phase duration. Coughing is a rhythmic process persisting also after the ending of stimulation, with lower intensity and prolongation. AspR and ExpR are solitary non-rhythmic respiratory processes and for their short durations they often do not disturbe the fictive cough attack [27,44,45]. Also prevention of proprioceptive reflexes in paralyzed animals eliminates some modification effects of airway reflexes, described in anaesthetized non-paralyzed animals [31,46] and their model [47], or chronic cough in patients [48,49].

***The expiration reflex*** induced by stimulation of the larynx is characterized by laryngo-constriction and prompt powerful expiratory effort without any inspiratory increase of actual lung volume [50,51]. The ExpR supports the CR to protect the airways and lungs from airborne and inhaled pathogens, allergens, aspirate and other irritants. Acute and chronic cough is a frequent symptom of respiratory tract irritations and disease, such as gastro - oesophageal reflux disease, asthma and COPD in humans [52]. Aspiration of pathogens present in foreign materials or secretions into the lungs, represents the most dangerous complication in patients with acute stroke, Parkinson’s disease, comatose states, long-term invasive artificial ventilation, nasogastric feeding, recurrent respiratory infections and dysphagia, resulting in *aspiration pneumonia*, particularly in the elderly [48,52-54]. Foreign materials and secretes containing viruses can be ejected by a strong expiratory effort up to 9 meters, and a patient can infect many co-passengers during airplane travel and can disseminate influenza and other respiratory infections to various destinations. Feeding through a nasogastric tube offers only limited protection against aspiration pneumonia, developing already after few days of illness in patients with dysphagia after acute stroke [55]. This may result from unwanted reflex aspiration provoked by nasopharyngeal stimulation with the catheter. Therefore, the down- and up- regulation of CR and ExpR may have a very positive, both preventive and therapeutic effects in patients. Testing the efficacy of CR in various pathological conditions can be useful for the assessment of the risk, as well as prevention and treatment of *aspiration pneumonia*[53-55]. Particularly the ExpR induced from the larynx, accompanied by glottal closure, is the main reflex that prevents aspiration [54,55]. Reliable ambulatory counter for cough have been developed for evaluation of cough count and intensity. Together with self-scoring evaluation of cough severity and impact on quality of life, it can be useful for diagnostic and antitussive therapy [56]. Similar short reflex expirations (ExpR) can be induced also by mechanical stimulation of the trachea in anaesthetized cats [57].

### Function of respiratory central pattern generator in airway reflexes

In defensive airway behaviours the laryngeal motoneurons are multifunctional in cats. During *fictive CR* the inspiratory laryngeal motoneurons (ILM) and expiratory laryngeal motoneurons (ELM), responsible for successive glottal dilation, closure, opening, and narrowing are active, parallel with the phrenic (PHR) or abdominal (ABD) motoneurons, contributing to the inspiratory or expiratory phase of CR, respectively. During the *fictive AspR* there is a strong activation not only of the PHR but also the ILM controlling the glottal dilators, and also the styloglossus muscle, providing tongue-back elevation. These activities provide an explosive inspiratory airflow during the AspR [58]. According to a recent computational biomechanical model many components of the raphe’-ponto-medullary system participate in the realization of the CR. This model based on successive transsections in rats and complementary calculations using 64 equations, resembles in vivo conditions. The DI caused by activation of PHR is accompanied by glottal dilation, provided by ILM. The expiratory phase of CR starts by activation of ELM and ABD motoneurons for glottal closure and production of strong expiratory effort, followed by activation of both ILM responsible for glottal opening, and ABD motoneurons providing rapid expiratory airflow. The intensity of second and further cough efforts gradually decreases, because of inactivation of high-pressure ILM, connected with successive lowering of peak lung volume, peak alveolar pressure, peak abdominal pressure and drive, resulting in lower peak expiratory flow [59].

Gradual transsection experiments in 4 weak old rats indicated, that the *respiratory CPG has 3 rhythmogenic capabilities,* realized by various neurons in 3 interacting structures. The intact ponto-medullary complex produces *a three-phase rhythm*, consisting of inspiration, post-inspiration and expiration. After elimination of the pons the PreBötC and the BötC of the medullary complex produce a *two-phase rhythm*, consisting from inspiration and expiration. After elimination of BötC the remaining **PreBötC** (proposed to be a “kernel” of respiratory rhythmogenesis), together with the rostral Ventral Respiratory Column (rVRC) produces *one-phase rhythm of rarer solitary inspirations,* influenced by hypercapnia [60], resembling gasping respiration.

A new computational model indicates that the regulation of respiratory rate and breathing pattern, provided by brainstem respiratory network, can be substantially influenced also by pulmonary and pontine feedback loops [61]. Similar model would be very useful for explanation of reconfiguration of breathing control by AspR during the stage of premortal gasping respiration, often resulting from asphyxia and providing autoresuscitation in both animals [6] and infants [7]. Mechanical stimulation of the NPh provokes AspR not only in severe hypoxia, manifesting by gasping, but also during eupnoea. The very similar character of AspR and gasping suggests that AspR transiently suppresses the brainstem mechanism responsible for neurogenesis of eupnoea, and activates those for gasping. The intensity of repeated AspR-es and accompanying inspiratory processes decrease only for ~4 cycles, but rapidly normalizes [62]. This special ability might allow use of AspR for experimental and model studies of autoresuscitation in general. It would be useful to explain the mechanism of reconfiguration of CPG provoking AspR by simple NPh stimulation, even in the stage of premortal gasping respiration in cats [35]. Practical application of powerful resuscitation potential of AspR in animals [19,31,35], and various revitalisation effects of its voluntary surrogate, represented by sniff in humans [40], seem to be very perspective.

### Cardio-respiratory and some resuscitation effects of airway reflexes

**Figure 4:**
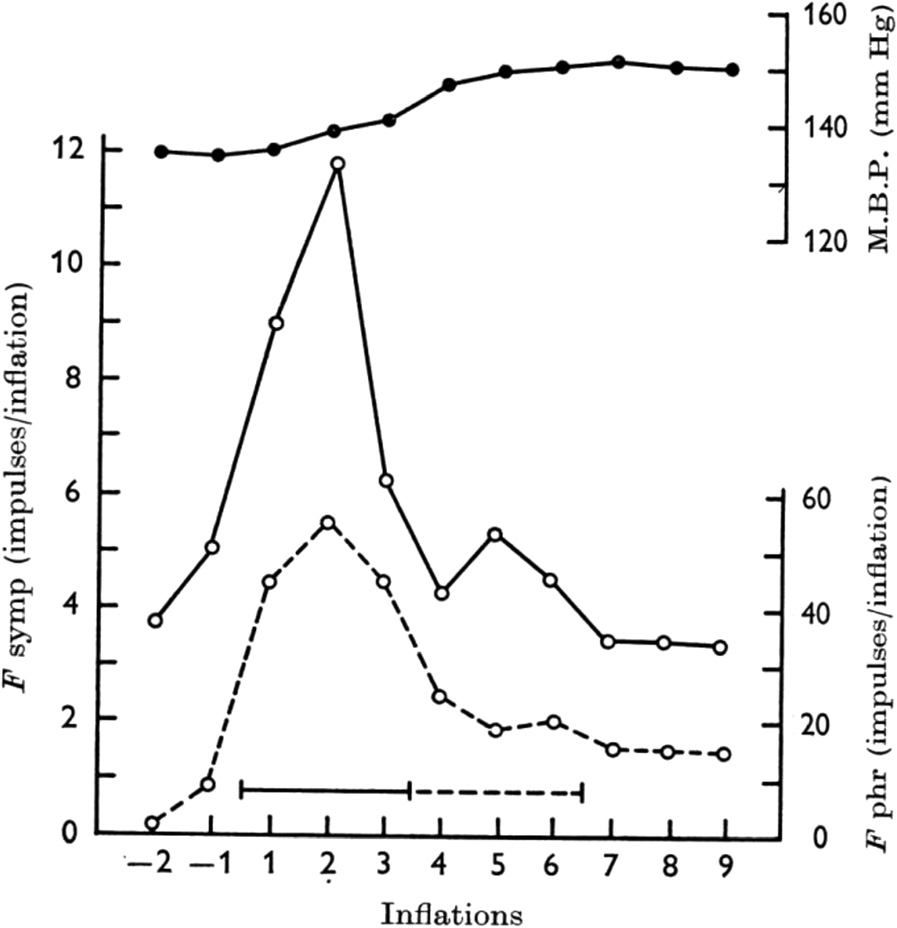
**Reaction of the phrenic and sympathetic nerve fibers and mean blood pressure on NPh stimulation in paralyzed cat.** Response of phrenic (--o--) and sympathetic (─o─) efferent fibres and of mean blood pressure (▬●▬) on nasopharyngeal stimulation averaged from five experiments on three fibre preparations in one paralyzed cat during artificial ventilation. Continuous line - common stimulation period in all experiments, interrupted line - stimulation finished in some experiments). Reproduced with permission from [18].

Recording of the activity of respiratory muscles and their neural connections, the pleural pressure and airflow parallely with BP and electrocardiogram (ECG), allows evaluation of the cardio-respiratory functions and their control mechanisms. They may reflect the character of pertinent changes in various vital functions, influencing the general state of the organism. The results of our systematic study indicated, that airway reflexes and particularly the AspR have similar resuscitation potential in animals as autoresuscitation by gasping. In patients with chronic heart failure hypoxia and not the frequency of apnoeic episodes causes the acute haemodynamic stress [63]. Hypoxia frequently causes life threatening nocturnal cardiac arrhythmias, which correlate with the severity of sleep apnoea [64], usually treated in patients with continuous positive airway pressure (CPAP). Hypoxic apnoea in cats, however, can be reversed by application of negative or even positive pressure or flow to isolated UA [24] as was indicated in Figure 3. This procedure provokes SIs of varying intensity, accompanied *usually by normalization of pathological cardiovascular activit*y (increase in HR and BP in collapsible states). This is followed by gradual reversal of cortical activity and recovery of spontaneous breathing [18,19]. Figure 4 illustrates that a very rapid and strong activity of the phrenic nerve, induced by stimulation of NPh in cats, responsible for the rapid and large inspiratory flow and volume of AspR, is accompanied by a strong activation of the sympathetic efferent fibers. This results in reflex tachycardia and severe vasoconstriction, causing a marked hypertensive reaction in paralyzed cats [18], demonstrating the revitalization effects of AspR.

Similar vasomotor reflexes can be provoked easily also by stimulation of cold receptors of the face and upper airways. Apnoea and bradycardia, followed usually by deep inspiration, peripheral vasoconstriction, and a systemic hypertensive reaction with redistribution of the blood to vital organs, are the main effects of this *diving reflex*. Stimulation of the orbito-frontal region of the face with cold water of 5°C for 5 s, reversed a supra-ventricular tachycardia from 300/min to 120/min in a neonate [65]. The diving reflex has a strong sympathetic component accompanying DI, in addition to other effects. Therefore, it proved to be very useful as a basic life support under collapsible states, to postpone and/or prevent an imminent loss of consciousness and a subsequent ischemic-hypoxic brain damage. From various methods of therapeutic hypothermia, widely used in polytraumatic patients the NPh balloon technique proved to be very effective [66]. In addition to direct cooling of caudal brain structures, also the NPh cold and mechanoreceptor stimulation may participate in the strong cerebral vasoconstriction, preventing ischemic and hypoxic brain damages.

Figure 3 illustrates a powerful resuscitation effect of AspR in a cat. Inhalation of CO_2_ after reactive transient hyperventilation evoked a gradual development of asphyxia and cardio-respiratory failure. It manifested by SIs and asphyxia culminating in apnoea, followed by repeated gasps. The asphyxia provoked severe bradycardia and extreme hypotension, accompanied by a progressive development of A-V blockade. In this agonal state each contact stimulation of the NPh mucosa elicited AspR-es, which were even stronger than the preceding repeated gasps and resulted in *cardio-respiratory resuscitation*[19]. Similar interesting case report published in 1991 indicated, that a present paroxysmal supraventricular tachycardia was terminated during *introduction of a nasogastric catheter* for gastric juice collection in a patient [67]. Recently, NPh aspiration by a catheter proved to be a treatment option for supraventricular paroxysmal arrhythmia in infants [68]. Clinical observations indicate, that also *hiccough attacks* can be easily terminated by introduction of a NPh catheter in patients [69]. Voluntary “*coughing on demand*”, proposed by Criley et al. [70] is frequently used for prevention and treatment of anaphylactic collapse, occurring during functional magnetic resonance (FMR) examination and in other collapsible states.

**Figure 5:**
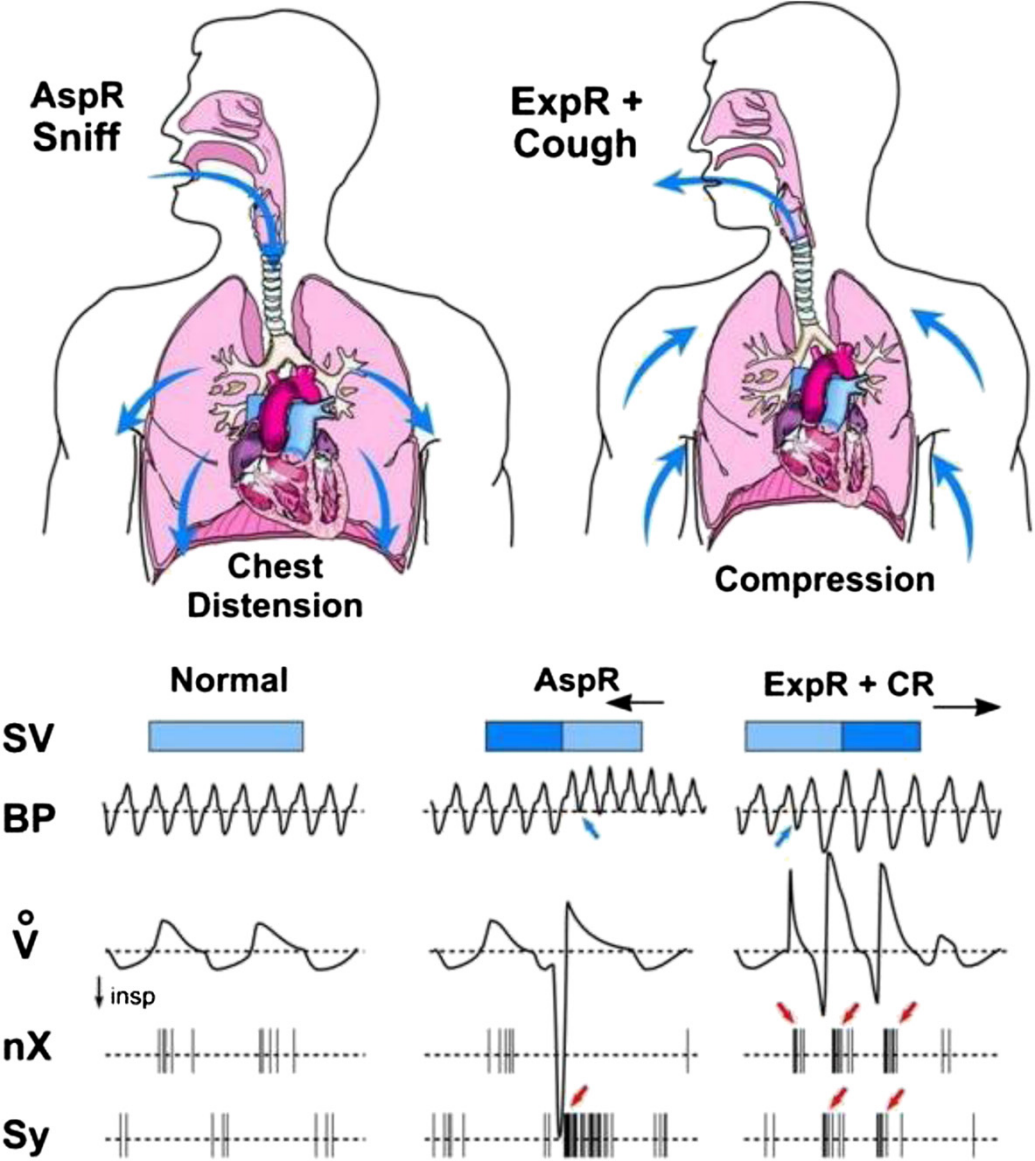
**Schematic illustration of the main effects of aspiration, expiration and cough reflexes in cats.** Compared to normal values, the sniff-like *aspiration reflex* (AspR), provoked by nasopharyngeal stimulation and characterized by rapid and strong gasp-like inspiratory flow (V’), manifests with distension of the chest and dilation of the cardiac cavities. This causes a decrease in the systolic volume (SV), due to retention of the blood in the heart. There is also a very strong activation of the sympathetic nerve (Sy), causing an increase in the blood pressure (BP) and a decrease in vagal activity (nX). On the contrary, the *expiration reflex* (ExpR), characterized by rapid and strong expiratory effort, manifesting with chest compression, which increases the next SV and BP and has a strong vagotonic effect. *The cough reflex* (CR) has even stronger effects than the ExpR, due to its deep inspiratory phase, adequately strengthening the successive expiratory effort. In addition, the preparatory initial inspiratory phase of cough is accompanied by a sympathetic activation, resulting in an increase in BP. Combined from results of our earlier publications [18,22,31,40].

Mechanical stimulations of NPh by a nylon fibre through a pharyngostomic opening, in anaesthetized *adult rats also evoked SIs*, very similar to AspR of cats. They were characterized by increase in tidal volume, airflow and minute volume, compared to control breaths. Such AspR-es provoked by mechanical stimulations, repeated in cycles of 16 s, significantly decreased the higher HR and the number of extrasystoles, caused by intraperitoneal application of aconitin [36]. AspR has various powerful *normalization and restorative effects*, which can inhibit different spastic events, such as bronchoconstriction and laryngospasm, as well as interrupts hypoxic apnoea, and inhibits the number and intensity of the cough efforts, at least in cats. Therefore, *AspR as a model of SIs* alone or combined with *forced expiration (ExpR)* or *prompt cough effort* (without preceding inspiration), can serve for reversal of many functional disorders, as indicated in our recent review [40]. The main effects of AspR, ExpR and CR are schematically illustrated in Figure 5.

### Perspective applications of airway reflexes for resuscitation

**Figure 6:**
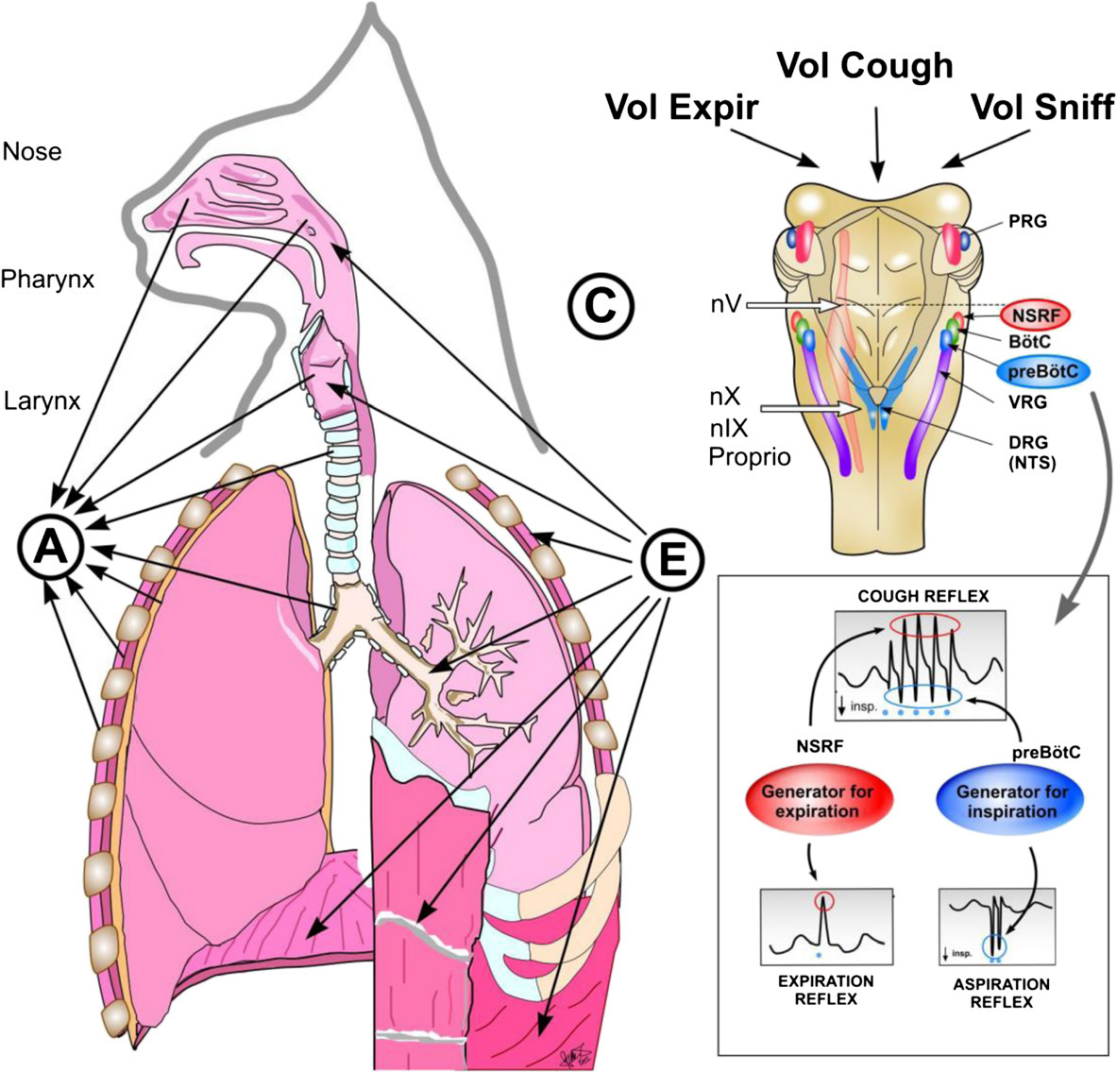
**Reflex arch and mechanisms of AspR, ExpR and CR and their voluntary counterparts.** Schematic illustration of the reflex arch and mechanisms of AspR, ExpR and CR and their voluntary counterparts. A- afferent roots: via trigeminal nerve from the upper part of nasal cavity for sniffing, via n. glosso-pharyngicus from the nasopharynx for AspR, via n. laryngicus sup. from the larynx for ExpR and via n. vagus from tracheal-bronchial region and proprioceptive afferents for CR. E- efferent roots: to inspiratory muscles (diaphragm and external intercostals) and to expiratory muscles (abdominal and internal intercostals) for ExpR and CR. C- central structures: “Generator for inspiration” in pre-Bötzinger complex for AspR, “generator for expiration” in NSRF- nucleus sub-retro-facialis for ExpR, and both generators for CR. Breathing manoeuvres allow voluntary performance of all three reflexes (voluntary sniffs, prompt expiration and sniff followed by strong expiration), reproduced with permission from our review [40].

The three analyzed airway reflexes very strongly activate the brainstem CPG of breathing, effectively *modifying the “central mechanisms” of various vital functions* by direct reflex action [31], or mediated by dense synaptic connections and various mediators [14]. Figure 6 schematically illustrates the reflex arch and central mechanisms of AspR, ExpR and CR. The effects of airway reflexes may promote normalization of hypo- and hyper-functional disorders, if not hindered by a presence of severe or fixed changes (e.g., acute stroke or recent myocardial infarction), both in animal experiments and probably also in human studies. *AspR* and *ExpR* in cats reversed several life-threatening disorders of functional character, manifesting as hypoxic apnoea termination and cardio-pulmonary-cerebral-resuscitation (CPCR) [19,31]. Therefore, the powerful AspR might influence positively also the disbalance between the excitatory and inhibitory cardio-respiratory impulses, deciding for survival or death in the patho-mechanism of SIDS, according to the hypothesis of Leiter and Böhm [71]. The powerful stimulatory effect of AspR, therefore, *might support the survival of dying animals, as well as SIDS infants and adult patients,* particularly in emergency situations and when there is no immediate health-care service on site. This effect results in a decrease of the number and intensity of disturbing cough efforts in anaesthetized cats [46]. In addition to strong gasp-like inspirations provoked by NPh mechanical stimulation, mediated by brainstem central control mechanisms participating in gasping, AspR is characterized also by reciprocal inhibition of expiratory activity.

Stimulation of the larynx both in cats and humans strongly activates the higher located brainstem expiratory mechanisms, causing laryngo-constriction and prompt expiratory effort without preceding inspiration. Similar short reflex expirations can be elicited also by tracheal stimulation in anaesthetized cats [57]. These efforts prevent intrusion of irritant materials and secretions into the lungs and provide their expulsion. Stimulation of the trachea-bronchial mucous membrane activates both the inspiratory and expiratory central mechanisms, provoking DI followed by powerful expiratory effort. The DI of CR provides venous return to the heart, and the successive strong expiratory effort supports brain perfusion, preventing and/or terminating various collapsible states [31]. Such *“on demand” provocations of CR might be very useful for study of CPCR i*n model experiments with dysrhythmias, including VF, particularly in animals, but supposedly also in clinical studies. Recent results indicate that already spontaneous gasps restored the cerebral blood flow (CBF) to 59% of the control values in animals. There was a very significant correlation of the CBF with the decreases in intrathoracic pressure during the inspiratory phase of gasps and with the increases of aortic pressure during their expiratory phase. Spontaneous gasps producing significant increases in the CBF during untreated cardiac arrest, confirmed the beneficial resuscitative effect of gasping during the cardiac arrest [72]. Experiences obtained in studies with CR in animals have relevance for human cough research [52]. The gradual decrease in intensity of successive cough efforts can be explained by HBEFR, which is very strong at the beginning of the expiratory period, reflecting the momentary relatively large lung volume [23].

### Long-term resuscitation effects of airway reflexes

Longer-lasting asphyxia may result in gradual decompensation of separate vital functions in general. Severe inspiratory resistive loading induces cardio-respiratory failure and not just an initial respiratory decompensation in anaesthetized rats [73]. A reduced respiratory activity results in increased ventilatory drive and evokes *an inactivity induced respiratory facilitation* with gradual restoration of breathing. Such breathing efforts were connected with an increase of mean BP, which proved to be the principal compensating factor in response to this cardio-respiratory failure, and supported generation of peak tracheal pressure [74,75]. Respiratory facilitation manifests also after spinal cord injury as crossed phrenic phenomenon [76]. Similar complex cardiovascular effects of resuscitation provided the required blood pressure increase, which started *the reversal of cardio-respiratory failure*, induced by severe hypoxia also in anaesthetized cats [31]. Similar facilitation was observed also during experimental VF in anaesthetized sheeps. Previous reflectoric movements of limbs caused that the thoraco-abdominal pump persisted to function for about 2 minutes in spite of total cardiac arrest, and caused synchronous changes in BP [77]. Coughing comprising initial DI, can allow both the maintenance of the venous return to the heart as well as brain perfusion, at least several minutes, which is sufficient for persistence of wakefulness, if needed. Therefore, voluntary rapid SIs each followed by prompt forced expiration (ExpR), might provide a powerful potential for *cardio-pulmonary- cerebral resuscitation* particularly in syncopal states, various collapses and even for prevention of sudden cardiac arrest [31,40]. Application of airway reflexes proved to reverse pathological situations (in animals 7 disorders) and (in humans 11 disorders), including asthma [78,79], indicated in a Table 1. However, there are still many open questions in this important topic.

**Table 1:** Main revitalisation effects of airway reflexes in animals (A) and airway reflexes or voluntary breathing manoeuvres in humans (H)

	**Reversal of pathological situations**	**Methods**
**A**	Interruption of bronchospasm in cats	NPh mechanical stimulation [18]
	Interruption of hypoxic apnoea in cats	NPh mechanical stimulation [19]
	Interruption of hypotension and bradycardia in cats	NPh mechanical stimulation [19]
	Interruption of asphyxia with collapse and A-V blockade in cats	NPh mechanical stimulation [19]
	Interruption of apneusis in cats	NPh mechanical stimulation [19]
	Interruption of aconitine arrhythmia in rats	NPh mechanical stimulation [36]
	Inhibition of excitability and rhythmicity of cough in cats	NPh mechanical stimulation [46]
**H**	Prevention of aspiration pneumonia in patients	Voluntary cough, ExpR [49]
	Interruption of paroxysmal supraventricular tachycardia in infant	Cold water on face - diving reflex [65]
	Interruption of paroxysmal supraventricular tachycardia in adult	NPh catheter [67]
	Interruption of paroxysmal supraventricular tachycardia in infants	NPh catheter [68]
	Prevention of brain damage in polytraumatic patients	Cold water in NPh balloon [66]
	Persisting breathing for 2 minutes during cardiac arrest in man	Reflex by previous limb exercise [77]
	Prevention of bronchospasm in asthmatics	Voluntary sniffs [78]
	Inhibition of capsaicin - induced cough in children with asthma	Voluntary sniffs [79]
	Treatment of hiccough in patients	NPh stimulation [69]
	PC communication and control of wheelchair by paraplegics	AspR and ExpR as binary symbols [40]
	Prevention of imminent collapse in patients	Voluntary „cough on demand” [70]

## Abbreviations

5HT: 5 hydroxy tryptamine; AspR: Aspiration reflex; A-V: Atrio-ventricular; BötzC: Bötzinger complex; CBF: Cerebral blood flow; CD: Cardiac death; CPG: Central pattern generator; CPCR: Cardio pulmonary cerebral resuscitation; CR: Cough reflex; DI: Deep inspiration; ECG: Electrocardiogram; EEG: Electroencephalogram; EMG: Electromyogram; ExpR: Expiration reflex; FMRI: Functional magnetic resonance imaging; HBEFR: Hering-Breuer expiration facilitating reflex; HBIR: Hering-Breuer inflation reflex; GG: Genio glossus muscle; NPh: Nasopharynx; NSRF: Nucleus sub-retro-facialis; Ppl: Pleural pressure; preBötzC: preBötzinger complex; RARs: Rapidly adapting receptors; SARs: Slowly adapting receptors; SI: Spasmodic inspiration; SIDS: Sudden infant death syndrome; UA: Upper airways; V’: Airflow; VF: Ventricular fibrillation; VT: Tidal volume.

## Competing interests

Zoltan Tomori and Viliam Donic were consultants of Nasophlex Slovakia, s.r.o. from January 2007 for testing of some effects of airway reflexes, which are in progress with use of 3 patent applications of a resuscitation device stimulating noninvasively the nasopharynx, sensitive points of the nasal filter and the external ear in sleep apnoea patients. All authors declare that they have no competing interests with the preparation of this paper. The patent “Resuscitation device and method for resuscitation” was approved for Australia no: 2006351860, for Canada no: 2,672,731, for Europe no: 12167089.3-2305, in progress for USA no: 2010/0042179 A1, and International no: WO 2008/072948-A1.

## Authors’ contributions

ZT developed the design of the experiments and the manuscript and VD, RB, SG and JJ equally helped him to study the mechanisms of the cough, aspiration and expiration reflexes and to complete the manuscript. All authors read and approved the final manuscript and informed the corresponding author with their consent.

## Authors’ information

Viliam Donic, Roman Benacka, Jan Jakus and Sona Gresova are co-authors.
